# Blood Pressure Variability and Severity of Early Prognosis in Patients with Acute Pontine Infarction

**DOI:** 10.1155/2020/1203546

**Published:** 2020-07-11

**Authors:** Xinsheng Han, Gaocai Zhang, Ning Liu, Hongyang Zhang, Jianke Xu, Miao Han, Yun Zhang, Yan Zhang, Li Chen

**Affiliations:** ^1^The Fifth Ward of Neurology Department, Kaifeng Central Hospital, Kaifeng 475000, Henan Province, China; ^2^The Child Neurology Department, Kaifeng Children's Hospital, Kaifeng 475000, Henan Province, China

## Abstract

**Background:**

Increased blood pressure (BP) variability may worsen the prognosis of stroke. This study aimed at investigating the association between BP variability and early functional prognosis in patients with pontine infarction.

**Methods:**

According to types of pontine infarction, all the 137 patients were divided into two groups: 70 patients with paramedian pontine infarction (PPI) and 67 patients with deep pontine infarction (DPI). Common risk factors, 24-hour continuous blood pressure monitoring data, and the coefficient of variation were collected after admission in the hospital. Functional outcomes were evaluated with modified Rankin scale (mRS) at 3 months after discharge (favorable outcome: mRS scores ≤ 2; poor outcome: mRS scores > 2).

**Results:**

The level of Glu, HbA1c, LDL, and NIHSS scores in the PPI group was significantly higher than that in the DPI group, and the concentration of blood uric acid was lower in the PPI group. Diastolic pressure in the PPI group is significantly higher than that in the DPI group, and coefficient of variation (CV) of systolic pressure in PPI is higher when compared with DPI ((88.77 ± 1.71) mmHg vs. (80.74 ± 1.31) mmHg; (11.54 ± 0.35) vs. (10.24 ± 0.25)). In multivariate analyses, the CV of systolic pressure, diastolic pressure, NIHSS scores, and the paramedian pontine infarction was independently associated with 3-month clinical outcome (OR = 1.94, 95% CI = 1.252–2.994, *P*=0.003; OR = 1.08, 95% CI = 1.002–1.166, *P*=0.04; OR = 1.58, 95% CI = 1.164–2.159, *P*=0.003; OR = 9.87, 95% CI = 1.045–32.193, *P*=0.04).

**Conclusion:**

In conclusion, increased 24-hour (BP) variability, NIHSS scores, and paramedian pontine were associated with early poor prognosis in patients with acute pontine infarction.

## 1. Introduction

Stroke, the third leading cause of death behind heart disease and cancer with its morbidity, disability, mortality, and high recurrence rate, has become one of the most serious diseases threatening human neurological health [1]. 85% of the stroke is ischemic stroke, and pontine infarction, the most common type of brainstem infarction, accounts for about 15% of posterior circulation strokes [2]. More and more risk factors such as age, gender, hyperlipidemia, and hypertension, are involved in the pathogenesis of cerebrovascular disease.

Hypertension is a serious risk factor for stroke [3] and may be an independent predictor of functional outcomes [4]. Although investing intensive research efforts, the researchers could found few effective treatments for acute stroke [5]. Elevated blood pressure (BP) in acute stroke associates with poor prognosis [6], so we can improve the outcomes by reducing the BP of patients with acute ischemic stroke. However, we cannot find a significant effect on the outcomes following the control of BP [7, 8]; instead, the incorrect use of antihypertensive drugs may increase the cerebral damage by reducing pressure-dependent cerebral perfusion to the ischemic penumbra, and Parati et al. observed that BP variability was strongly prognostic for morbidity and mortality [9].

By impairing the structure of vascular endothelial function, unsteadiness of BP variability could lead to atherosclerosis, and the formation of atherosclerotic plaques, BP variability, in turn, leads to vascular atherosclerosis or other vascular diseases [10–12]. BP variability may be the independent risk factor for arterial atherosclerosis in patients with acute stroke [12]. BP variability was associated with hemispheric stroke location, and the patients with stroke in the left hemisphere had more unstable BP variability [12, 13]. However, there is uncertainty about the relationship between BP variability, lesion location, and prognosis in acute pontine infarction.

We hypothesized that BP variability and localization of pontine infarction would contribute to poor neurological outcomes in acute pontine infarction. The aim of this study was to evaluate the influence of BP variability on pontine infarction and early prognosis based on the infarct location in the pontine infarction.

## 2. Materials and Methods

### 2.1. Patients

The study included clinical data of 137 consecutive patients admitted for acute pontine infarction and hospitalized between December 2016 and September 2018 in Kaifeng Central Hospital. Patients were enrolled in this study when they fulfilled the following criteria: (1) nonatherosclerotic factors such as cardiogenic cerebral embolism, vasculitis and dissection, and other unknown etiologies, and (2) no history of malignancy, brain tumor, intracerebral hemorrhage, severe cardiac failure, severe hepatic failure, severe renal failure, renal hypertension, or endocrine diseases, such as primary hyperaldosteronism or pheochromocytoma. Clinical early outcomes were evaluated using the modified Rankin Scale (mRS) at 3 months after admission. This study was approved by the Ethics Committee of the Kaifeng Central Hospital. Written informed consent was obtained from all patients prior to their enrolment in this study. Each patient was evaluated by at least one neurologist. The diagnosis was based on clinical presentation and confirmed by magnetic resonance imaging (MRI) findings of the brain and carried out during the first 48 h after admission. Neurologists, unaware of the patient's blood pressure (BP), reviewed the MRI scans to characterize the pontine infarction type. All the cases were divided into two groups according to stroke location: paramedian pontine infarction (PPI) (70 patients: 47 males and 23 females) and deep pontine infarction (DPI) (67 patients: 47 males and 20 females). Demographic data and medical history were recorded, as well as whether the patient had hypertension prior to the stroke.

### 2.2. Measurements of BP

The BP was measured by the ambulatory blood pressure monitoring (CB-1805-B, Wuxi, China) [14], which was validated for accuracy by BSI Product Services of UK in 2009. The BP was measured in the nonparalytic arm. Systolic pressure and diastolic pressure were autonomically measured every 20 minutes for 08:00–20:00 and 30 minutes for 20:00–08:00. The raw data were used to obtain 24-hour mean systolic pressure and diastolic pressure, and the indices of BP variability such as SD and coefficient of variation (CV (%) = SD × 100/mean value) during the 24-hour period were calculated.

### 2.3. Neuroimaging Procedures

Cerebral infarctions and their locations were evaluated using a 1.5 T MRI scanner (MAGNETOM Avanto; Siemens Medical Systems, Erlangen, Germany) on the day of admission. Acute ischemic stroke was diagnosed on diffusion-weighed imaging (DWI : TR = 6,000 ms, TE = 88 ms, and slice thickness = 5 mm).

### 2.4. Statistical Analysis

All variables were expressed as means, or numbers (percentage). Analysis of variance or the Mann–Whitney *U* test was performed for continuous variables, and the *χ*2 test or Fisher's exact test was performed for categorical variables. Binary logistic regression models were established to investigate risk factors contributing to poor outcome at 3 months after discharge. Values of *P* < 0.05 were considered significant. SPSS statistical software version 23 (SPSS Inc., Chicago, IL, USA) was used for statistical analysis.

## 3. Results

### 3.1. Background and Medical History

A total of 137 patients were included in the study, and no patients died during the follow-up period. The medical characteristics of all the patients are shown in Table 1. The percentage of patients with history of diabetes in the PPI group was significantly higher than that in the DPI group.

### 3.2. Analysis of Risk Factors and Therapies

The common risk factors are shown in Table 2. Our findings suggested that the level of Glu, HbA1c, LDL, and NIHSS scores in the PPI group was significantly higher than that in the DPI group (*P* < 0.05), whereas, in contrast with PPI group, the concentration of blood uric acid was lower than that in the DPI group (*P* < 0.05). There was no significant difference in HDL, cholesterol, urea, creatinine, and homocysteine between the two groups (*P* > 0.05). All patients received basic therapy including antiplatelets, lipid-lowering medications, and improving the function of mitochondria, and nearly 10% of them received thrombolytic therapy, but we did not find significant difference in the therapies between the two groups (*P* > 0.05).

### 3.3. Blood Pressure Variability in Acute Pontine Infarction

In order to study the blood pressure variability between different groups of patients, 24-hour dynamic blood pressure monitoring was performed. Our study showed that diastolic pressure in the PPI group is significantly higher than that in the DPI group. CV of systolic pressure in PPI is higher when compared with DPI. However, we could not find any difference in SD of systolic and diastolic BP between the two groups (Table 3).

### 3.4. Basilar Artery (BA) Stenosis in the PPI and DPI Groups

In our study, we observed the relationship between the location of pontine infarction and BA stenosis by magnetic resonance angiography (MRA). There were 51 patients having BA stenosis in PPI, only 9 in the DPI group. We found that the stenosis rate of BA in PPI was significantly higher than that in DPI by chi-squared test (Table 4).

### 3.5. Binary Logistic Analysis of the Relationship between BP, Other Variables, and the Poor Outcome at 3 months after Discharge

The OR and 95% CI were calculated after adjusting for the following variables: glycated hemoglobin, LDL, CV of systolic pressure, diastolic pressure, uric acid, infarction location, and NIHSS scores, for they have difference between the two groups (*P* < 0.1). Our finding suggested that CV of systolic pressure, diastolic pressure, infarction location, and NIHSS scores was directly associated with the poor outcome at 3 months (Table 5).

## 4. Discussion

The present study demonstrated that the systolic BP variability and diastolic BP were associated with 3-month clinical outcome. And the effect was independent of common risk factors such as LDL, age, and gender. Further analysis showed that the patients in the PPI group had poor outcome, suggesting lesion location is most strongly correlated with neurological outcomes of patients with pontine infarction. It has been suggested that BP variability plays an important role in the development of vascular events and may result in poor outcome [15–17]; to our knowledge, no previous reports have investigated the relationship between BP variability and early outcomes in patients with pontine infarction.

It has been previously reported that age, history of diabetes mellitus, arterial stenosis, and high serum urea have been associated with prognosis [18–21]. Among these factors, the associations with arterial stenosis and mRS score were reproduced in the present study. In line with the previous research, our findings indicated that the morbidity of basilar artery stenosis in the PPI group is higher than that in the DPI group, accompanying poor neurologic function.

Previous studies have reported that systolic BP variability in the acute phase of ischemic stroke is associated with neurological lesion, poor outcome, and even early death [22, 23]. However, short-term BP variability was not a predictor of early outcomes in acute ischemic stroke [24]. In the present study, the early prognosis was worse in patients with PPI than that with DPI, accompanying lower systolic BP variability. R. K. Poortvliet et al. [25] illustrated that diastolic BP variability is significantly associated with stroke events and mortality, which is consistent with this research, and we found the diastolic BP in the PPI group is significantly higher than in the DPI group, and diastolic BP is a strong predictor for the neurological impairment, but we cannot find the difference of diastolic BP variability between the two groups. Further studies should elucidate the associations between the diastolic BP variability and the early outcomes.

Previous studies have reported the location of pontine infarctions is associated with the poor outcomes [26–28], and we found that the patients in PPI group are serious. It may be the large infarct volume itself resulting poor early outcome. And, during acute ischemic stroke, hyperglycemia may worsen the clinical outcome of patients with acute ischemic nonlacunar stroke, but not lacunar stroke [29]. In our study, there was no association between hyperglycemia and early prognosis, which was consistent with the other paper, considering 83.4% of the lesions in the pons were infarcts caused by atheromatous occlusion or lipohyalinotic degeneration [30].

Some limitations need to be noted in the present study. Firstly, being a single-center study, the universality of the study may be limited. This is an observational study, so the results may not clearly state the causal relationship between BP variability and early neurological outcome. Secondly, the associations between BP variability and the neurological outcome may differ according to different cause of each case, the sample size of our study was insufficient, and we cannot examine heterogeneity among the potential causes for the insufficient of the sample size in our study. In addition, we did not measure infarct volume and did not calculate the circadian rhythm of BP in this study, and further study is needed to verify our findings.

Despite the above limitations, our results suggested that there has a relationship between BP variability and early prognosis in acute pontine infarction for the first time. These results provided a potential modifiable risk factor for the therapy of pontine infarction. Further research was needed to investigate the causal relation between BP variability and early prognosis.

## Figures and Tables

**Table 1:** Demographic data and medical history of study participants, number (%).

Variables	PPI (*n* = 70)	DPI (*n* = 67)	*χ* ^2^ value	*P* value
Age (years)	61.81 ± 1.48	63.49 ± 1.15		0.4
Male (%)	67.1	70.1	0.1	0.7
Tobacco smoking (%)	47.1	41.8	0.4	0.5
Hypertension (%)	78.6	85.1	1.0	0.3
CCB (%)	54.5	51.8	0.2	0.9
ACEI (%)	45.5	48.2	0.2	0.9
Diabetes mellitus^*∗*^ (%)	37.1	19.4	5.3	0.02
History of stroke (%)	38.6	31.3	0.8	0.4
Statin (%)	100.0	100.0		0.9^#^

Data are shown for the patients' demographics. ^*∗*^*P* < 0.05; ^#^Fisher test. CCB, calcium channel antagonist; ACEL, angiotensin-converting enzyme inhibitor.

**Table 2:** The common risk factors and therapies of 136 patients at admission.

Variables	PPI (*n* = 70)	DPI (*n* = 67)	*P* value
Glucose	7.4 ± 0.4	6.3 ± 0.3	0.02
Glycated hemoglobin	7.0 ± 0.3	6.2 ± 0.2	0.01
LDL	2.7 ± 0.1	2.4 ± 0.1	0.03
HDL	1.1 ± 0.03	1.1 ± 0.03	0.3
Cholesterol	4.5 ± 0.1	4.3 ± 0.1	0.4
Triglyceride	1.4 ± 0.1	1.5 ± 0.1	0.5
Apolipoprotein A1	1.3 ± 0.03	1.3 ± 0.03	0.8
Apolipoprotein B	0.8 ± 0.02	0.9 ± 0.04	0.5
Lipoprotein	236.0 ± 31.3	197.5 ± 26.2	0.4
Creatinine	69.5 ± 2.1	71.0 ± 2.4	0.6
Urea	7.0 ± 1.0	6.7 ± 1.0	0.8
Uric acid	293.8 ± 10.5	325.4 ± 11.3	0.04
C-reactive protein	4.9 ± 2.5	3.5 ± 0.5	0.6
D-dimer	0.3 ± 0.02	0.3 ± 0.03	0.3
Fibrinogen	2.7 ± 0.1	2.7 ± 0.1	0.9
WBC	7.0 ± 0.2	7.3 ± 0.3	0.4
Non-HDL	3.3 ± 0.1	3.2 ± 0.1	0.5
Hemoglobin	140.4 ± 2.4	143.8 ± 1.8	0.3
Hcy	18.2 ± 2.5	21.6 ± 2.3	0.3
NIHSS score	4.9 ± 0.4	2.3 ± 0.3	<0.01
Thrombolytic therapy (*n*, %)	11 (15.7%)	7 (10.4%)	0.4
Acute antihypertensive treatment (*n*, %)	18 (25.7)	22 (32.8)	0.4

LDL, low-density lipoprotein; HDL, high-density lipoprotein; Hcy, homocysteine. Data are shown for the patients' demographics. ^*∗*^*P* < 0.05. NIHSS, National Institutes of Health Stroke Scale.

**Table 3:** Differences in BP variability between patients in the DPI and PPI groups.

Variables	PPI (*n* = 70)	DPI (*n* = 67)	*P* value
Systolic pressure (mmHg)	145.7 ± 2.1	148.9 ± 2.4	0.3
Diastolic pressure (mmHg)	88.8 ± 1.7	80.7 ± 1.3	0.01
Mean BP (mmHg)	108.1 ± 1.6	110.9 ± 1.8	0.3
CV of systolic pressure (%)	11.5 ± 0.4	10.2 ± 0.3	0.03
CV of diastolic pressure (%)	15.3 ± 0.3	15.3 ± 0.4	0.8
SD of systolic pressure	15.3 ± 0.4	15.4 ± 0.4	0.9
SD of diastolic pressure	11.7 ± 0.3	12.3 ± 0.4	0.2

Data are shown for the feature of blood pressure. ^*∗*^*P* < 0.05.

**Table 4:** Analysis of BA stenosis in the PPI and DPI groups.

Groups	Cases	BA stenosis	
		Yes	No
PPI	70	51	19
DPI	67	9	58
*χ* ^2^ value		49.1	
*P* value		<0.01	

**Table 5:** Binary logistic analysis of the relationship between PPI, CV of systolic pressure, diastolic pressure, and the poor outcome at 3 months.

Variable	Wald value	*P* value	OR	95.0% CI
PPI (+)	4.0	0.04	9.9	1.1–32.2
CV of systolic pressure (%)	8.8	0.003	1.9	1.3–3.0
Diastolic pressure (mmHg)	4.0	0.04	1.1	1.0–1.2
NIHSS score	8.5	0.003	1.6	1.2–2.2

Binary logistic regression for the poor outcome at 3 months. ORs and their CIs were obtained by logistic regression analysis simultaneously adjusted for glycated hemoglobin, LDL, CV of systolic pressure, diastolic pressure, uric acid, infarction location, uric acid, and NIHSS score. NIHSS, National Institutes of Health Stroke Scale. PPI(+) = 1, PPI(−) = 0.

## Data Availability

The data used to support the findings of this study are available from the corresponding author upon request.
